# Leptin Inhibits the Proliferation of Vascular Smooth Muscle Cells Induced by Angiotensin II through Nitric Oxide-Dependent Mechanisms

**DOI:** 10.1155/2010/105489

**Published:** 2010-06-01

**Authors:** Amaia Rodríguez, Javier Gómez-Ambrosi, Victoria Catalán, Ana Fortuño, Gema Frühbeck

**Affiliations:** ^1^Metabolic Research Laboratory, University of Navarra, 31008 Pamplona, Spain; ^2^CIBER Fisiopatología de la Obesidad y Nutrición, Instituto de Salud Carlos III, Spain; ^3^Division of Cardiovascular Sciences, Center for Applied Medical Research, University of Navarra, 31008 Pamplona, Spain; ^4^Department of Endocrinology, Clínica Universidad de Navarra, 31008 Pamplona, Spain

## Abstract

*Objective*. This study was designed to investigate whether leptin modifies angiotensin (Ang) II-induced proliferation of aortic vascular smooth muscle cells (VSMCs) from 10-week-old male Wistar and spontaneously hypertensive rats (SHR), and the possible role of nitric oxide (NO). *Methods*. NO and NO synthase (NOS) activity were assessed by the Griess and ^3^H-arginine/citrulline conversion assays, respectively. Inducible NOS (iNOS) and NADPH oxidase subutnit Nox2 expression was determined by Western-blot. The proliferative responses to Ang II were evaluated through enzymatic methods. *Results*. Leptin inhibited the Ang II-induced proliferative response of VSMCs from control rats. This inhibitory effect of leptin was abolished by NOS inhibitor, NMMA, and iNOS selective inhibitor, L-NIL, and was not observed in leptin receptor-deficient *fa/fa* rats. SHR showed increased serum leptin concentrations and lipid peroxidation. Despite a similar leptin-induced iNOS up-regulation, VSMCs from SHR showed an impaired NOS activity and NO production induced by leptin, and an increased basal Nox2 expression. The inhibitory effect of leptin on Ang II-induced VSMC proliferation was attenuated. *Conclusion*. Leptin blocks the proliferative response to Ang II through NO-dependent mechanisms. The attenuation of this inhibitory effect of leptin in spontaneous hypertension appears to be due to a reduced NO bioavailability in VSMCs.

## 1. Introduction

Hypertension is associated with structural changes in blood vessels known as “vascular remodelling” that include an altered proliferation, hypertrophy, migration, and apoptosis of vascular smooth muscle cells (VSMCs), together with an increased extracellular matrix abundance [1]. Angiotensin (Ang II) constitutes one of the main factors involved in vascular remodelling during the onset of hypertension [1]. Angiotensin II exerts pleiotropic actions on the vasculature, such as vasoconstriction, VSMC migration, proliferation and hypertrophy, increased extracellular matrix formation, and activation of NAD(P)H oxidases [1, 2]. Through these actions Ang II promotes vascular inflammation as well as endothelial dysfunction and structural remodelling.

Leptin, the obesity gene (*ob*) product, participates in the control of body weight by regulating food intake and energy expenditure [3, 4]. In addition to the maintenance of energy homeostasis, leptin induces a balanced effect on the control of blood pressure (BP) with a pressor response attributable to sympathetic activation via the central nervous system and a depressor response due to a direct effect of leptin on peripheral tissues [5]. Leptin increases the vasomotor sympathetic activity through the activation of leptin receptors (OB-R) in the ventromedial and dorsomedial hypothalamic regions [6]. On the other hand, leptin exerts a direct vasodilation through different mechanisms, which include the release of endothelial nitric oxide (NO) in the aorta and coronary arteries [7–9] and endothelium-derived hyperpolarizing factor (EDHF) in mesenteric arteries [8, 10], as well as the inhibition of the Ang II-induced calcium increase and vasoconstriction in the smooth muscle layer of the aorta via NO [11]. A further mechanism whereby leptin decreases BP is related to the induction of natriuresis and diuresis at the tubular level through NO-dependent mechanisms [12, 13]. Increased circulating concentrations of leptin are found in hypertensive animal models [14, 15] and humans [16, 17], suggesting a possible link between hyperleptinemia, and cardiovascular dysfunction in hypertension. In this respect, it has recently been reported that the beneficial vascular, renal and cardiac responses induced by leptin are impaired in hypertensive rats [10, 12, 14, 15].

Leptin has been suggested to participate in vascular remodelling, since it induces the proliferation of rat aortic VSMCs [18] and promotes neointimal growth of VSMCs after injury in mice [19]. Nonetheless, these data are not univocal, given that other authors have reported that leptin inhibits cell growth of human VSMCs [20]. These contradictory observations raise some doubts as regards the potential involvement of leptin in vascular remodelling. The present study was designed to examine the effect of leptin on basal and Ang II-induced proliferation of aortic VSMCs obtained from normotensive Wistar rats and age-matched, spontaneously hypertensive rats (SHR). Some experiments were performed upon VSMCs obtained from Zucker *fa/fa *rats to confirm whether the effects of leptin are mediated via OB-R. To gain further insight into the potential role of NO in the proliferative response induced by leptin, the effect of leptin on NO production, NO synthase (NOS) activity and inducible NOS (iNOS) expression was measured directly in VSMCs. Moreover, to further corroborate the participation of NO in the vascular actions of leptin, the effect of the N^G^-monomethyl-L-arginine (NMMA), a nonselective inhibitor of NOS, and L-N^6^-(1-iminoethyl)-lysine (L-NIL), a selective inhibitor of iNOS, on the inhibitory effect of leptin on the Ang II-induced proliferation of VSMC of the aorta was analyzed.

## 2. Materials and Methods

### 2.1. Animals

Age-matched (10-week-old) male normotensive Wistar (breading house of the University of Navarra), SHR and leptin receptor-deficient Zucker *fa/fa *rats (Harlan, Barcelona, Spain) were used in the present study. Rats were maintained under controlled conditions of room temperature (RT) (20 ± 2°C), relative humidity (50 ± 10%), ventilation (at least 15 complete changes of air/h), and artificial light-dark cycle (lights on from 08:00 a.m.–08:00 p.m.). Animals had free access to tap water and fed *ad libitum* with an isoenergetic (13.39 MJ/kg), isoproteic (14%) rodent maintenance diet containing 0.13% sodium (2014S Teklad Global 14% Protein Rodent Maintenance Diet, Harlan). All experimental procedures conformed to the European Guidelines for the Care and Use of Laboratory Animals (Directive 86/609/EEC) and were approved by the Ethical Committee for Animal Experimentation of the University of Navarra (036/03). After regular overnight feeding, rats were sacrificed by decapitation in a nonfasted state, since fasting has been shown to reduce circulating concentrations of leptin [4]. Blood samples were immediately collected, and sera were obtained by cold centrifugation (4°C) at 700* g* for 15 minutes. The thoracic aorta was carefully excised, dissected out, and processed for each study.

### 2.2. Blood Measurements

Serum glucose concentrations were measured using a sensitive-automatic glucose sensor (Ascensia Elite, Bayer, Barcelona, Spain). Serum concentrations of triglycerides, total cholesterol (Infinity, Thermo Electron Corporation, Melbourne, Australia), and free fatty acids (FFA) (WAKO Chemicals, GmbH, Neuss, Germany) were measured by enzymatic methods, using available commercial kits. Insulin and leptin were determined by ELISA (Crystal Chem, Inc., Chicago, IL, USA). Intra- and interassay coefficients of variation for measurements of insulin and leptin were 3.5% and 6.3%, respectively, for the former, and 5.4% and 6.9%, for the latter. Lipid peroxidation, as an indicator of oxidative stress, was estimated by the measurement of thiobarbituric acid reactive substances (TBARS) in serum as previously described by Conti et al. [21] with some modifications. Serum malondialdehyde (MDA), the best-known specific TBARS, was used as indicator of lipid peroxidation and oxidative stress. Five *μ*L of serum samples or standard MDA (Sigma, St. Louis, MO, USA) were mixed with 120 *μ*L of diethyl thiobarbituric acid (DETBA) 10 mmol/L and vortexed for 5 seconds. The reaction mixture was then incubated at 95°C for 60 minutes. After cooling to room temperature (RT) for 5 minutes, DETBA-MDA adducts were extracted in 360 *μ*L  *n*-butanol (Panreac, Barcelona, Spain) vortexing for 1 minute and centrifuged at 1,600* g* for 10 minutes at RT. Then, the chromophore of the DETBA-MDA adduct was quantified in 200 *μ*L of the upper butanol phase by fluorescence emission at 535 nm with an excitation at 590 nm. MDA equivalents (TBARS) were quantified using a calibration curve prepared using MDA standard working solutions.

### 2.3. Isolation of Vascular Smooth Muscle Cells

Primary VSMCs were obtained from the thoracic aorta by the tissue explants method, as previously described [11, 15]. Briefly, the smooth muscle tissue was longitudinally opened and cut in small pieces that were grown in plastic 6-well plates and maintained at 37°C in a humidified incubator with an atmosphere of 95% air, 5%  CO_2_. Tissue explants were cultured in Dulbecco's modified Eagle's medium (DMEM) containing 20% fetal bovine serum (FBS) (Life Technologies, Inc., Gaithersburg, MD, USA) and antibiotic-antimycotic products (10,000 U/mL penicillin G sodium, 10,000 *μ*g/mL streptomycin sulfate, and 25 *μ*g/mL amphotericin B as Fungizone in 0.85% saline) (Life Technologies). The medium was changed initially after 24 hours, and then every 2-3 days. After about 8–10 days, when cells had formed a confluent monolayer, they were harvested by addition of 0.05% trypsin, and the culture was continued up to 4–6 passages using DMEM containing 10% FBS.

### 2.4. Cell Proliferation Assay

Cell proliferation of VSMCs was measured using the CellTiter 96 Aqueous One Solution cell proliferation assay (Promega, Charbonnier, France), according to the manufacturer's instructions. VSMCs were plated in 96-well plate (3,500 cell per well) and incubated for 24 hours in DMEM containing 10% FBS. Quiescence was induced by incubating the cells in DMEM containing 0.1% FBS for 48 hours. Serum-deprived VSMCs were stimulated for 72 hours with different concentrations of Ang II (0.1–1,000 nmol/L) (Sigma) in order to obtain a concentration-response curve for the determination of the pD2 value. In a second subset of experiments, cells were incubated with different concentrations of leptin (0.1–100 nmol/L) (PreproTech EC, Inc., Rocky Hill, NJ, USA) for 72 hours in the absence or presence of Ang II (100 nmol/L). In a third subset of experiments, cells were stimulated with leptin (10 nmol/L) for 72 hours in the presence of Ang II (100 nmol/L) and NMMA (10 *μ*mol/L) (Sigma) or L-NIL (10 *μ*mol/L) (Sigma). The concentration of leptin as well as the pharmacological NOS inhibitors to carry out the experiments was chosen on the basis of prior experiments performed in our laboratory [11, 15]. Following the cell treatment, 20 *μ*L of CellTiter 96 Aqueous One Solution were added to each well and the plate was incubated in the darkness at 37°C for 4 hours. Optical densities were measured at 490 nm using a microplate reader (Sunrise, Tecan, Germany). Proliferative values were expressed as percentage of proliferation of treated cells compared to basal proliferation of unstimulated cells.

### 2.5. Western-Blot Analyses

Quiescent VSMCs were stimulated for 30 minutes with leptin (10 nmol/L). At different times of the stimulation (0, 10, 20, and 30 minutes), cells were harvested and homogenized in ice-cold lysis buffer (0.1% SDS, 1% Triton X-100, 5 mM EDTA*·*2H_2_O, 1 M Tris-HCl, 150 mM NaCl, 1% sodium deoxycholate, pH 7.40) supplemented with a protease inhibitor cocktail (Complete Mini-EDTA free, Roche, Mannheim, Germany). Lysates were centrifuged at 16,000* g *at 4°C for 15 minutes. Total protein concentrations were determined by the Bradford assay [22], using bovine serum albumin (BSA) (Sigma) as standard [23]. Thirty micrograms of total protein were diluted in loading buffer 4X (20%  *β*-mercaptoethanol, 40 mmol/L dithiothreitol, 8% SDS, 40% glycerol, 0.016% bromophenol blue, 200 mmol/L Tris-HCl, pH 6.80) and heated for 10 minutes at 100°C. Samples were run out in 8% SDS-PAGE, subsequently transferred to nitrocellulose membranes (Bio-Rad Laboratories, Inc., Hercules, CA, USA) and blocked in Tris-buffered saline (10 mmol/L Tris-HCl, 150 mmol/L NaCl, pH 8.00) with 0.05% Tween 20 (TBS-T) containing 5% nonfat dry milk for 1 hour at RT. Blots were then incubated overnight at 4°C with rabbit polyclonal anti-Akt1, rabbit polyclonal anti-phospho-(Thr^308^)-Akt (Upstate, Lake Placid, NY, USA), rabbit polyclonal anti-STAT3, rabbit polyclonal anti-phospho-(Tyr^705^)-STAT3 (Santa Cruz Biotechnology, Inc., Santa Cruz, CA, USA), mouse monoclonal anti-iNOS (BD Transduction Laboratories, San Jose, CA, USA), rabbit polyclonal anti Nox2/gp91phox (Abcam, Cambridge, UK), or murine monoclonal anti-*β*-actin (Sigma) antibodies. The antigen-antibody complexes were visualized using peroxidase-conjugated antirabbit or antimouse antibodies (1 : 5,000) and the enhanced chemiluminescence ECL detection system (Amersham Biosciences, Buckinghamshire, UK). The intensity of the bands was determined by densitometric analysis and normalised with *β*-actin density values.

### 2.6. Evaluation of NO Production and NOS Activity

Quiescent VSMCs were stimulated during 30 minutes with leptin (10 nmol/L) in the presence or absence of NMMA (10 *μ*mol/L) or L-NIL (10 *μ*mol/L). One sample per assay was used to obtain control responses in the presence of solvent. Samples of the culture media were collected at different times (0, 10, 20 and 30 minutes) for the measurement of nitrates and nitrites ([NO_x_]), as an index of NO production, with a commercial kit (Cayman Chemical, Ann Arbor, MI, USA) based on the Griess reaction following the manufacturer's protocol. The intra- and inter-assay coefficients of variation were 3.3% and 6.5%, respectively. Stimulated cells were harvested and homogenised in a lysis buffer (25 mmol/L Tris, 1 mmol/L EDTA, 1 mmol/L EGTA; pH 7.40) supplemented with a protease inhibitor cocktail (Roche) for the determination of NOS activity. The protein content of the homogenates was determined by the method of Bradford [22]. NOS activity was measured by the L-[^3^H]arginine to L-[^3^H]citrulline conversion assay, using a commercial kit (Stratagene, La Jolla, CA, USA). The intra- and inter-assay coefficients of variation were 6.3% and 9.1%, respectively. Briefly, samples of 20 *μ*g of protein were incubated at room temperature (RT) for 1 hour in the reaction buffer [25 mmol/L Tris-HCl (pH 7.40), 3 *μ*mol/L tetrahydrobiopterin, 1 *μ*mol/L FADH, 1 *μ*mol/L FMNH_2_, 1 mmol/L NADPH, 0.6 mmol/L CaCl_2_] supplemented with L-[^3^H]arginine (1 *μ*Ci/*μ*L) (Amersham Biosciences). L-[^3^H]citrulline was quantified by using a scintillation counter (Wallac 1409 DSA, PerkinElmer, Inc., Barcelona, Spain). All assays were performed in duplicate.

### 2.7. Statistical Analysis

Data are presented as mean ± standard error of the mean (SEM). Concentration-response curves were fitted by nonlinear regression, the concentration giving 50% of the maximal response (EC_50_) was determined, and the pD_2_ was calculated as −log EC_50_ (mol/L). Statistical differences among mean values were determined using the two-way ANOVA, one-way ANOVA followed by Dunnett's *t* test, or the Student's *t* test, where appropriate. A *P* value <  .05 was considered statistically significant. Analyses were performed by the SPSS/Windows version 15.0.1 software (SPSS Inc., Chicago, IL, USA).

## 3. Results

### 3.1. Metabolic Profile and Serum Leptin Concentrations

General characteristics of the carbohydrate and lipid metabolism of experimental animals are shown in Table 1. SHR were heavier (*P* < .001) and exhibited higher serum glucose (*P* < .01) and insulin (*P* < .001) concentrations than age-matched Wistar rats. Serum triglycerides and total cholesterol were also increased (*P* < .05 and *P* < .01, resp.) in SHR, compared to Wistar rats. The circulating concentrations of leptin were increased (*P* < .05) in the SHR group. A positive correlation between serum leptin levels and body weight (*r *= 0.67, *P* < .0001) was found. The serum levels of TBARS, as the index of oxidative stress, were significantly (*P* < .05) increased in SHR compared to control rats. 

### 3.2. Effect of Leptin on Ang II-Induced Proliferative Response in VSMCs

**Figure 1:**
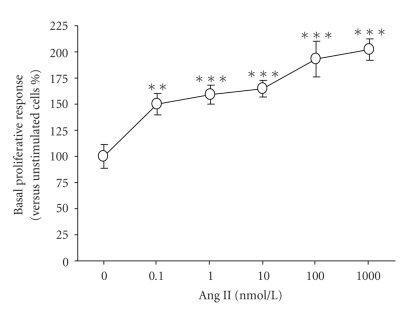
Concentration-response curve of the proliferation induced by angiotensin (Ang) II in aortic vascular smooth muscle cells (VSMCs) obtained from Wistar rats. Values are the mean ± SEM (*n *= 10–15). Differences between groups were analysed by one-way ANOVA followed by Dunnet's test. **P < .01, ***P < .001 versus control response in unstimulated cells.

Ang II elicited a concentration-dependent (*P* < .00001) increase in the proliferation of aortic VSMCs obtained from Wistar rats (pD_2_ = 9.1 ± 0.6) (Figure 1). A concentration of Ang II 100 nmol/L, inducing a proliferative response of 193 ± 17% compared to basal proliferation, was chosen for subsequent experiments. 

**Figure 2:**
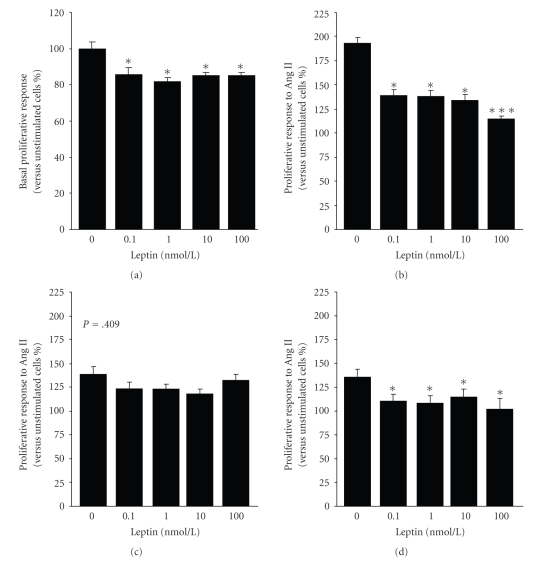
Effect of leptin on basal and Ang II-induced proliferation of aortic VSMCs. Aortic VSMCs obtained from Wistar rats were incubated for 72 hours with increasing concentrations of leptin (0.1–100 nmol/L) in the absence (a) or presence (b) of Ang II (100 nmol/l), and the proliferative response was measured using a tetrazolium dye (MTT)-based proliferation assay. Effect of leptin on Ang II (100 nmol/l)-induced proliferation in VSMCs obtained from the aorta of leptin receptor-deficient Zucker *fa/fa *rats (c) and spontaneously hypertensive rats (SHR). Values are the mean ± SEM (*n* = 40). Differences between groups were analysed by one-way ANOVA followed by Dunnet's test. *P <  .05, ***P <  .001 versus control response in unstimulated cells (a) or to Ang II (b, c).

All the tested leptin concentrations significantly inhibited (*P* < .05) the basal proliferation of aortic VSMCs from Wistar rats (Figure 2(a)). Moreover, leptin induced a decrease (*P* < .01) in Ang II-induced proliferative response in VSMCs from Wistar rats (Figure 2(b)). To test that the inhibitory effect of leptin is mediated via its binding to leptin receptors, the experiments were also performed in VSMCs obtained from Zucker *fa/fa *rats, a genetic model of leptin receptor resistance. As earlier reported by other authors [24, 25], Zucker *fa/fa *rats were severely obese, and showed hyperglycaemia, hyperinsulinemia, hyperlipidemia, and hyperleptinemia (Table 2). No inhibitory effect of leptin (*P* = .409) was observed on Ang II-induced proliferation in VSMCs obtained from the aorta of Zucker *fa/fa* rats (Figure 2(c)).

To determine whether this vascular action of leptin may be altered in hypertension, we assessed the effect of leptin on Ang II-induced proliferative response in aortic VSMCs from SHR rats. Although leptin was able to inhibit (*P* < .01) the Ang II-induced proliferation in VSMCs from SHR (Figure 2(d)), the reduction of the response to Ang II was lower than that of control Wistar rats in all tested concentrations of leptin (0.1 nmol/L, 18 ± 6% versus 28 ± 4%; 1 nmol/L, 17 ± 5% versus 28 ± 3% versus 17 ± 5%; 10 nmol/L, 15 ± 6% versus 31 ± 3%; 100 nmol/l, 41 ± 2% versus 24 ± 8%, resp.).

### 3.3. Effect of Leptin on Ang II-Induced Proliferation of VSMCs in the Presence of NOS Inhibitors

**Figure 3:**
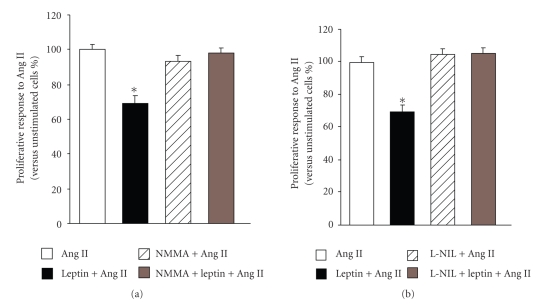
Impact of NOS inhibitors on the inhibitory effect of leptin on Ang II-induced proliferation of aortic VSMCs. The coincubation with both NOS inhibitor, NMMA (10 *μ*mol/l), (a) and the selective iNOS inhibitor, L-NIL (10 *μ*mol/l), (b) blunted the inhibitory effect of leptin (10 nmol/l) on the Ang II (100 nmol/l)-induced proliferative response in aortic vascular smooth muscle cells (VSMCs) from Wistar rats. Data are expressed as mean ± SEM (*n*=40). Differences between groups were analysed by two-way ANOVA. In case of interaction between factors (leptin treatment and NOS inhibitors), differences between groups were analysed by one-way ANOVA followed by Dunnet's test. *P < .05, **P <  .01 versus control response to Ang II in the absence of inhibitors.

Our group previously described that leptin induces the synthesis of NO through the activation of iNOS in VSMCs [11]. The effect of leptin on the Ang II-induced proliferative response of aortic VSMCs obtained from Wistar rats was reexamined in the presence of the NOS inhibitor, NMMA, or the iNOS selective inhibitor, L-NIL. The concentration of leptin 10 nmol/L, reducing by ~15% the basal proliferation and by ~30% the Ang II-induced proliferation in aortic VSMCs, was chosen to carry out these experiments. Both NOS inhibitors completely abolished the inhibitory effect of leptin on the Ang II-mediated proliferation (Figure 3). Moreover, the presence of NMMA or L-NIL blunted the inhibition of basal proliferation induced by leptin (105 ± 1% and 107 ± 2% versus 85 ± 2% mg, resp.). Basal and Ang II-induced proliferation of aortic VSMCs was not affected by the presence of NOS inhibitors.

### 3.4. Impaired NOS Activity and NO Production in VSMCs in Hypertensive Rats

**Figure 4:**
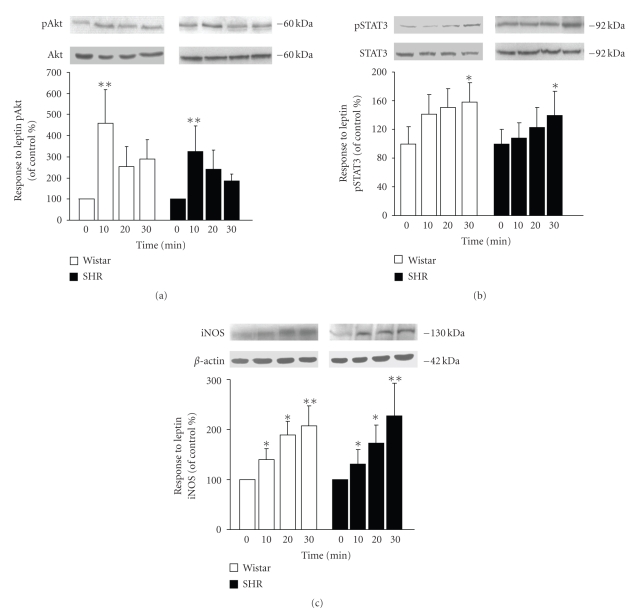
Effect of leptin activation of Akt and STAT3 and iNOS expression in aortic VSMCs. Bar graphs show the differences in the time course of Akt (a) and STAT3 (b) activation/phosphorylation as well as iNOS (c) protein expression in leptin (10 nmol/L)-stimulated aortic VSMCs from Wistar and SHR. Data are expressed as mean ± SEM (*n* = 10). Differences between groups were analysed by two-way ANOVA. In case of interaction between factors (strain and time of leptin stimulation), differences between groups were analysed by one-way ANOVA followed by Dunnet's test *P < .05, **P < .01 versus unstimulated cells.

**Figure 5:**
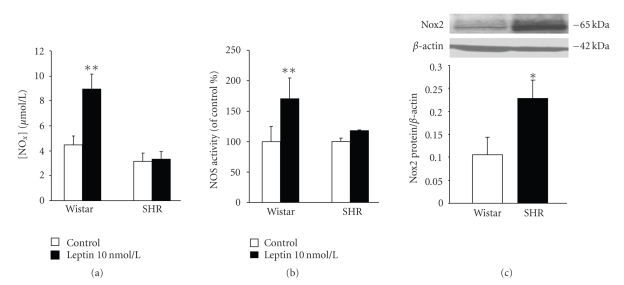
Impaired NO production and NOS activity and increased NADPH oxidase expression in aortic VSMCs from hypertensive rats. Bar graphs show (a) the accumulation of NO_x_ in the culture media and (b) the NOS activity of aortic VSMCs from control Wistar rats and spontaneously hypertensive rats (SHR) stimulated with leptin (10 nmol/l) for 30 minutes. (c) Basal expression of Nox2, a subunit of the NADPH oxidase, in VSMCs from control Wistar rats and SHR. Data are expressed as mean ± SEM (*n* = 10). Differences between groups were analysed by two-way ANOVA (a, b) or Student's *t* test (c). In case of interaction between factors (strain and leptin treatment), differences between groups were analysed by one-way ANOVA followed by Dunnet's test *P < .05, **P <  .01 versus control response in unstimulated cells (a, b) or VSMCs from control rats (c).

The activation of the JAK2/STAT3 and PI3K/Akt pathways constitutes an early step for the up-regulation of iNOS induced by leptin [11, 26, 27]. The ability of leptin to trigger JAK2/STAT3 and PI3K/Akt was examined by the degree of phosphorylation/activation of the downstream molecules STAT3 and Akt after leptin treatment in VSMCs from Wistar rats and SHR. Leptin activated the phosphorylation of STAT3 in a time-dependent manner, whereas a maximal phosphorylation of Akt was observed after 10 minutes of leptin stimulation with attenuation of the phosphorylation thereafter (Figures 4(a) and 4(b)). No differences between VSMCs from Wistar and SHR were found for the activation/phosphorylation of Akt and STAT3. Accordingly, leptin induced a significant increase in iNOS expression in aortic VSMCs from Wistar and SHR (Figure 4(c)). Nevertheless, the ability of leptin to induce NO production and NOS activity was impaired in aortic VSMCs obtained from SHR (Figures 5(a) and 5(b)). It is well known that enhanced production of NO-scavenger substances such as reactive oxygen species (ROS) under spontaneous hypertension is involved in reducing NO bioavailability [2]. Thus, we compared the basal expression of Nox2, a subunit of the ROS-generating NADPH oxidase, in VSMCs from control Wistar rats and SHR. The protein levels of Nox2 were significantly (*P* < .05) increased in VSMCs from hypertensive rats (Figure 5(c)).

## 4. Discussion

The smooth muscle layer represents an important target for the vascular effects of leptin [11, 15]. This adipokine decreases passive wall tension and Ang II-induced vasoconstriction operating directly on VSMCs [11]. Despite the growing evidence supporting the depressor action of leptin on blood vessels, the role of leptin on vascular remodelling remains unclear [18–20]. Thus, the present study has further explored the mechanisms whereby leptin participates in the proliferation of VSMCs, a crucial process involved in vascular remodelling.

Our results show that leptin inhibits the basal proliferation of aortic VSMCs in Wistar rats, which is in concordance with findings reported by Bohlen and colleagues using human aortic VSMCs [20]. Moreover, we show, for the first time, that leptin inhibits the Ang II-induced cell growth of VSMCs. To test directly whether this inhibitory effect is dependent on leptin signalling, the experiments were also performed in VSMCs obtained from the aorta of Zucker *fa/fa *rats, an animal model with a missense mutation in the leptin receptor gene (OB-R^269gln→pro^) that results in both a reduced affinity for leptin and reduced signal transduction capability [24, 25]. As a result of this genetic leptin receptor resistance, Zucker rats show severe metabolic alterations, including severe obesity, hyperglycemia, hyperinsulinemia, insulin resistance, and hypogonadism [24, 25]. This animal model of leptin resistance, that is, the obese Zucker *fa/fa *rats, also shows hypogonadism that further aggravates the obese phenotype, since leptin can regulate the expression and secretion of gonadotropins, and the hypothalamic-pituitary-gonadal axis is closely associated to food intake, body weight, and fat distribution [28]. In the present study, our findings showed the lack of effect of leptin on Ang II-induced proliferation in aortic VSMCs from Zucker rats, suggesting that functional leptin receptors are required for this vascular effect of the hormone.

A functional relation between leptin and NO has been established in blood vessels [7, 8, 11, 29]. Frühbeck showed that intravenous administration of leptin in rats with autonomic blockade induces a systemic vasodilation that is associated with an increase of serum [NO_*x*_] and reversed with N*ω*-nitro-L-arginine methyl ester [7]. Further studies have shown that leptin induces an endothelial-dependent vasodilation by activating a PI 3-kinase-independent Akt-endothelial NOS (eNOS) phosphorylation pathway [29, 30]. Moreover, leptin treatment in vivo has been shown to reverse the endothelial dysfunction of leptin-deficient obese (*ob/ob*) mice by increasing NO bioavailability in vessels [31]. This adipokine decreases passive wall tension and Ang II-induced vasoconstriction by up-regulating iNOS through mechanisms involving JAK2/STAT3 and PI3K/Akt pathways in VSMCs [11]. The ability of leptin to induce iNOS gene expression has been shown in several cell types, such as murine J774A.1 macrophages, rat adipocytes, human primary chondrocytes and ATDC5 cells, C6 glioma cell line, and human OA cartilage [26, 27, 32–34]. Our findings showed that the inhibitory effect of leptin on Ang II-induced cell growth of VSMCs is completely prevented by an iNOS inhibitor. Previous data reported by our group provided evidence that the depressor action of leptin in the smooth muscle layer of the aorta takes place by reducing the vasoconstrictor potential of Ang II through NO-dependent mechanisms [11]. Similar findings of hypotensive effects of leptin via NO have been reported in rat myocardium [35], kidneys[13], endothelium of conduit vessels (aorta) [8, 29, 30], and resistance vessels (mesenteric and coronary arteries) [8, 9]. Taken together, these data support the notion that NO represents a key mediator of the cardiovascular effects of leptin.

SHR constitute a well-known model of essential hypertension that becomes hypertensive at an early stage (4–6 weeks of age) [36]. Early vascular remodelling experienced by the aorta of SHR leads to a reduced contractility in vitro and, probably, to vascular rigidity in vivo [2, 15]. Overactivation of the renin-angiotensin system constitutes an important contributor to the vascular remodelling associated with the onset of hypertension in SHR [1]. Touyz and colleagues [37] reported that Ang II concentration dependently increased the ^3^H tymidine incorporation in VSMCs, as an index of synthesis of DNA and cell proliferation, with enhanced responsiveness in VSMCs from SHR compared to control rats. In the present study, SHR showed features of the human metabolic syndrome, such as overweight, hyperglycaemia, hyperinsulinemia, insulin resistance, dyslipidemia, and increased circulating concentrations of leptin, which confirm data reported by our group and others [12, 38]. Moreover, our results show that aortic VSMCs from SHR are less responsive to the inhibitory effect of leptin on Ang II-induced proliferation. These findings are in agreement with other studies reporting an impairment of the depressor actions of leptin (or leptin resistance) under spontaneous hypertension in rat myocardium [14], mesenteric arteries [10], aorta [15], and kidneys [12]. Interestingly, we found that, despite a similar activation of JAK2/STAT3 and PI3K/Akt and increased expression of iNOS than that observed in normotensive rats, VSMCs from SHR showed an impaired NOS activity and NO production induced by leptin as well as higher basal Nox2 expression, a subunit of the reactive oxygen species (ROS)-producing NADPH oxidases. It is well known that endothelial dysfunction in SHR is characterized by a reduced synthesis and release of endothelium-derived relaxing factors, such as NO and/or an enhanced production of reactive oxygen species (ROS), which scavenge NO within vessels to reduce its biological half-life [2]. In VSMCs, Ang II reportedly increased the ROS-generating enzymes NADPH oxidases and ROS function as important intra- and intercellular second messengers to modulate many downstream signalling molecules, such as protein tyrosine phosphatases (PTPs), protein tyrosine kinases, transcription factors, mitogen-activated protein kinases (MAPKs), and ion channels, leading to VSMCs growth and migration [2]. In the present study, our data showed an increased systemic oxidative stress as well as higher expression levels in VSMCs of Nox2 in hypertensive rats. Together, it could be speculated that, in the setting of hypertension, the antiproliferative effects of leptin are overridden by the effects of Ang II, despite the hyperleptinemia. Among the different mechanisms that may underlie this finding, the role of Ang II-induced production of ROS could be important in experimental hypertension.

In conclusion, our results provide evidence that leptin constitutes a negative modulator of vascular remodelling. This statement is supported by findings reported herein: (a) leptin inhibits the basal and Ang II-induced proliferative response of VSMCs through NO-dependent mechanisms; (b) the lack of effect of leptin on Ang II-stimulated proliferation in VSMCs obtained from leptin receptor-deficient Zucker *fa/fa *rats provides evidence that functional leptin receptors (OB-R) are required for this inhibition; (c) the impairment of the inhibitory effect of leptin on Ang II-induced proliferation of VSMC from SHR appears to be a consequence of a reduced NO biodisponibility due to an increased expression of NADPH oxidases. Therefore, hyperleptinemia may arise as a compensatory mechanism to overcome vascular leptin resistance in SHR.

## Figures and Tables

**Table 1:** Metabolic characteristics of normotensive and hypertensive animals.

Determination	Wistar rats (*n *= 14)	SHR (*n *= 28)	*P* value
Body weight (g)	283.6 ± 9.4	303.3 ± 2.6	**.001**
Free fatty acids (mg/dL)	20.8 ± 1.7	20.3 ± 0.9	.776
Triglycerides (mg/dL)	105.3 ± 19.2	129.5 ± 6.7	**.05**
Total cholesterol (mg/dL)	113.5 ± 6.4	134.1 ± 3.2	**.002**
Glucose (mg/dL)	133.4 ± 0.7	205.5 ± 0.5	**.002**
Insulin (ng/mL)	1.3 ± 0.3	3.0 ± 0.3	**.001**
Leptin (ng/mL)	2.5 ± 0.1	3.1 ± 0.1	**.05**
TBARS (*μ*mol/L)	1.4 ± 0.2	2.2 ± 0.2	**.025**

SHR, spontaneously hypertensive rats; TBARS, thiobarbituric acid reactive substances. Values presented as the mean ± SEM. Differences between groups were analysed by Student's *t-*test. Bold values are statistically significant *P* values among groups.

**Table 2:** Metabolic characteristics of Zucker *fa/fa *rats.

Determination	Wistar rats (*n *= 14)	Zucker *fa/fa *rats (*n *= 10)	*P* value
Body weight (g)	283.6 ± 9.4	403.1 ± 4.9	**.00001**
Free fatty acids (mg/dL)	20.8 ± 1.7	15.7 ± 2.1	.081
Triglycerides (mg/dL)	105.3 ± 19.2	285.1 ± 16.6	**.00001**
Total cholesterol (mg/dL)	113.5 ± 6.4	131.4 ± 2.1	**.05**
Glucose (mg/dL)	133.4 ± 0.7	166.8 ± 1.1	**.05**
Insulin (ng/mL)	1.3 ± 0.3	10.8 ± 0.4	**.00001**
Leptin (ng/mL)	2.5 ± 0.1	49.8 ± 0.3	**.00001**

Values presented as the mean ± SEM. Differences between groups were analysed by Student's *t *test. Bold values are statistically significant *P* values among groups.
